# Elevated Expression of *TGFB1* in PBMCs Is Associated with Intracranial Aneurysm Formation, but *TGFB3* Expression Implicated Rupture

**DOI:** 10.3390/biomedicines13061273

**Published:** 2025-05-22

**Authors:** Kinga Sutkowska, Olga Martyna Koper-Lenkiewicz, Marta Żebrowska-Nawrocka, Marta Jakoniuk, Tomasz Łysoń, Marzena Tylicka, Ewa Balcerczak, Joanna Matowicka-Karna, Joanna Kamińska

**Affiliations:** 1 Department of Clinical Laboratory Diagnostics, Clinical Hospital of the Medical University of Bialystok, 15A Jerzego Waszyngtona St., 15-269 Bialystok, Poland; olga.koper-lenkiewicz@umb.edu.pl (O.M.K.-L.); joanna.matowicka-karna@umb.edu.pl (J.M.-K.); 2 Department of Clinical Laboratory Diagnostics, Medical University of Bialystok, 15A Jerzego Waszyngtona St., 15-269 Bialystok, Poland; 3 Department of Pharmaceutical Biochemistry and Molecular Diagnostics, Medical University of Lodz, 1 Muszynskiego St., 90-151 Lodz, Poland; marta.zebrowska@umed.lodz.pl (M.Ż.-N.); ewa.balcerczak@umed.lodz.pl (E.B.); 4 Laboratory of Molecular Diagnostics, BRaIn Laboratories, Medical University of Lodz, 4 Czechoslowacka St., 92-216 Lodz, Poland; 5 Department of Invasive Neurology, Medical University of Bialystok, 24A M. Sklodowskiej-Curie St., 15-276 Bialystok, Poland; jakoniukmarta@gmail.com (M.J.); tomasz.lyson@umb.edu.pl (T.Ł.); 6 Department of Neurosurgery, Clinical Hospital of the Medical University of Bialystok, 24A M. Sklodowskiej-Curie St., 15-276 Bialystok, Poland; 7 Department of Biophysics, Medical University of Bialystok, 2A Adama Mickiewicza St., 15-089 Bialystok, Poland; marzena.tylicka@umb.edu.pl

**Keywords:** intracranial aneurysm (IA), peripheral blood mononuclear cells (PBMCs), transforming growth factor beta (TGF-β), risk factors, ruptured intracranial aneurysm (RIA), unruptured intracranial aneurysm (UIA)

## Abstract

**Introduction:** The transforming growth factor beta (TGF-β) signaling pathway plays a critical role in cellular processes, including maintaining vascular integrity and regulating vascular remodeling. Aneurysm rupture is associated with pathological changes in the arterial wall. **Aims:** We aimed to investigate the gene expression of transforming growth factors (*TGFB1*, *TGFB2*, *TGFB3*) in peripheral blood mononuclear cells (PBMCs) isolated from the blood of patients with unruptured intracranial aneurysms (UIAs) and ruptured intracranial aneurysms (RIAs), and from a control group. Additionally, we evaluated serum levels of TGF-β1, TGF-β2, and TGF-β3 and analyzed their associations with various risk factors, including sex, age, aneurysm size, number, shape, smoking, and hypertension. **Materials and Methods:** The study group consisted of patients diagnosed with intracranial aneurysms (IAs) who were eligible for embolization at the Department of Neurosurgery, Clinical Hospital of the Medical University of Bialystok. The control group consisted of healthy volunteers, recruited from the employees of the Clinical Hospital of the Medical University of Bialystok. Expression levels were assessed using quantitative real-time polymerase chain reaction techniques in PBMCs. Serum concentrations of TGF-β isoforms were evaluated using a multiplexed bead-based immunoassay. **Results:** Among 32 patients, 24 had unruptured intracranial aneurysms (UIAs), including 18 women and 6 men, while 8 presented with ruptured intracranial aneurysms (RIAs), evenly distributed between women and men (4 each). The mean age of the patients was 53 years (range: 24–71 years). The control group consisted of 20 healthy volunteers, 14 females and 6 males, with a mean age of 51 years (range: 24–71 years). The expression of *TGFB1* was significantly higher in the IA versus C group, but *TGFB3* expression was significantly higher in the RIA versus C group. The serum level of TGF-β1 and TGF-β3 was significantly higher in the RIA versus UIA group. Serum TGF-β1 levels were higher in men and individuals < 60 years of age. Positive correlations were observed between serum TGF-β1, TGF-β3 and aneurysm size, with significantly higher TGF-β3 levels in patients with giant aneurysms. **Conclusions:** Our study highlights the distinct roles of *TGFB1* and *TGFB3* in aneurysm pathophysiology, identifying *TGFB1* as a molecular contributor to aneurysm formation and *TGFB3* with rupture. Increased serum TGF-β1 and TGF-β3 concentrations could serve as promising noninvasive parameters for assessing the risk of aneurysm rupture. Further research with larger cohorts is needed to define cut-off values and validate the method, enabling the use of blood TGF-β levels as a tool for clinical decision-making.

## 1. Introduction

Contemporary advances in medical imaging technology have significantly enhanced the detection of intracranial aneurysms (IAs), which are characterized by pathological dilatation of the walls of cerebral arteries. Although most incidentally detected aneurysms remain asymptomatic, their rupture can result in subarachnoid hemorrhage (SAH), a condition with a mortality rate of 35% to 50% [1,2]. Given these life-threatening consequences, many patients choose therapeutic interventions that, while potentially beneficial, carry substantial risks. Therefore, the search for parameters that could assist in identifying aneurysms with a high risk of rupture is crucial. Such parameters would enable the qualification of patients for endovascular or neurosurgical procedures only if absolutely necessary, while minimizing the risks associated with such interventions in cases where there is no indication for them [3].

Intracranial arteries are composed of three layers: the tunica intima, tunica media, and tunica externa. The tunica intima is composed of endothelial cells (ECs), subendothelial connective tissue, and the internal elastic lamina (IEL). The tunica media primarily contains smooth muscle cells (SMCs), while the tunica externa, which is rich in collagen fibers and elastin, also contains nerves and fibroblasts, forming the external elastic lamina (EEL) [4]. Numerous studies have indicated that aneurysm rupture is associated with a series of pathological changes in the arterial wall [3,5,6,7,8,9,10,11,12].

In recent years, the transforming growth factor beta (TGF-β) signaling pathway has garnered particular interest among researchers due to its critical role in cellular processes. TGF-β, homodimeric protein, is a multifaceted growth factor produced by a variety of cell types, including ECs, T lymphocytes, macrophages, fibroblasts, platelets, and astrocytes [13,14,15]. There are three major isoforms of TGF-β: TGF-β1, TGF-β2, and TGF-β3, and three primary receptor types (TβR-I, TβR-II, and TβR-III) [16,17,18,19]. The role of TGF-β is complex and context-dependent, varying according to the type of target cell. TGF-β is a critical regulator of vascular development, where it significantly contributes to the reinforcement of the vascular wall. Additionally, it mediates the vascular response to injury by promoting repair mechanisms. TGF-β upregulates the expression of connective tissue growth factor, a key molecule involved in various cellular processes such as proliferation, adhesion, migration, and the synthesis of extracellular matrix components, thereby supporting vascular remodeling and repair mechanisms [5,14]. TGF-β induces the expression of collagen and elastin genes. It also promotes vascular smooth muscle cell (SMC) growth, differentiation, proliferation, and inhibition [14].

Recent studies suggest that TGF-β may be strongly associated with intracranial aneurysms (IAs) [3,20,21,22]. Cerebral aneurysm pathogenesis involves intricate vascular remodeling driven by endothelial dysfunction, smooth muscle cell (SMC) phenotypic switching, and chronic inflammation. The researchers suggest that TGF-β can enhance vascular structure and integrity while also triggering metalloproteinase activation, SMCs degeneration and apoptosis, inflammatory cell infiltration, promotion of inflammatory processes, and oxidative stress, all of which may contribute to aneurysm development and rupture [5,23,24,25,26,27,28,29,30,31]. The significance of TGF-β in vascular pathology has been confirmed by genetic studies demonstrating a link between mutations in the TGF-β pathway in Loeys–Dietz syndrome, which manifests as severe vascular abnormalities [32,33,34,35]. Experiments in animal models have shown that the loss of TGF-β pathway genes in ECs leads to significant vascular defects [36,37]. The hypothesis regarding the role of TGF-β in the pathogenesis of IA has been supported by studies documenting increased TGF-β expression in aneurysm walls [37,38].

The involvement of TGF-β in vascular repair and pathological vascular remodeling justifies our investigation into its role as a key mediator in aneurysm formation. Additionally, understanding TGF-β’s function may help estimate the risk of aneurysm rupture, even in cases where the aneurysm remains stable for years, especially since the literature data suggest that the mechanisms underlying cerebral aneurysm formation and rupture differ significantly [25]. Therefore, to gain a deeper insight into the role of TGF-β factors in the formation and rupture of IA, we evaluated the gene expression of all three transforming growth factors (*TGFB1*, *TGFB2*, *TGFB3*) in peripheral blood mononuclear cells (PBMCs) isolated from the blood of patients with unruptured intracranial aneurysms (UIAs) and ruptured intracranial aneurysms (RIAs), comparing these findings with a control group. PBMCs comprise a heterogeneous population of immune cells, including T cells, B cells, natural killer cells, monocytes, and dendritic cells, which are implicated in aneurysm formation and rupture [39]. The search for circulating parameters that could assist in identifying aneurysms with a high risk of rupture is crucial. Therefore we investigated the serum levels of TGF-β1, TGF-β2, and TGF-β3, exploring their associations with various risk factors such as sex, age, aneurysm size, count, shape, smoking, and hypertension for intracranial aneurysm growth and rupture.

## 2. Material and Methods

### 2.1. Subjects

The research adhered to the guidelines outlined in the Declaration of Helsinki, and the protocol was approved by the Bioethics Human Research Committee at the Medical University of Bialystok (Permission No. APK.002.203.2024). Prior to their involvement in the study, all participants provided informed consent for inclusion.

The study group consisted of patients with intracranial aneurysms (IAs) who were qualified for aneurysm embolization based on computed tomography angiography at the Department of Neurosurgery, Clinical Hospital of the Medical University of Bialystok. The control group (C) consisted of healthy volunteers, recruited from the employees of the Clinical Hospital of the Medical University of Bialystok. Exclusion criteria were stringent for both groups, including the presence of neurodegenerative conditions such as multiple sclerosis, a history of neuro-infections or brain tumor, recent surgery or major trauma in the preceding months, or the administration of antibiotics, anti-inflammatory drugs, or corticosteroids within the past month.

### 2.2. Sample Collection and Storage

For patients in the RIA group, blood was collected within approximately 1 h of hospital admission; in both UIA and RIA groups, blood was collected at the beginning of the aneurysm embolization procedure. Whole arterial blood samples were collected in 5.5 mL tubes without anticoagulant (catalog #: 03.1397.001 S-Monovette, SARSTEDT, Newton, NC, USA) to obtain serum, and in 9 mL K3E (1.6 mg EDTA/mL) tubes (catalog #:02.1066.001, S-Monovette, SARSTEDT, Newton, NC, USA) for peripheral blood mononuclear cell (PBMCs) isolation. Serum separation was performed by centrifuging the blood at 1000× *g* for 20 min, within 30 min of venipuncture. The PBMC isolation procedure was also initiated within 30 min of collection using Histopaque (catalog #: 10771, Sigma Life Science, St. Louis, MO, USA). Samples were subsequently stored at −80 °C until analysis, and experiments were conducted immediately after thawing.

### 2.3. Peripheral Blood Mononuclear Cell (PBMCs) Isolation Using Density Gradient Centrifugation with Ficoll Histopaque

PBMCs isolation was performed on 32 samples from patients in the IA group and 18 samples from the control group (C) according to the following procedure:

1. Measured 3 mL of Ficoll Histopaque into each of three 10 mL centrifuge tubes.
2. Gently layered 3 mL of blood on top of the Ficoll Histopaque using a 1 mL automatic pipette, layering very slowly to ensure that the blood and Ficoll Histopaque remained as two distinct layers.
3. Centrifuged the tubes (without delay) for 30 min at 400× *g* at 20 °C.
4. Aspirated the whitish buffy coat (approximately 1.5 mL) of PBMCs that formed at the interface between the Ficoll Histopaque and medium.
5. Washed the PBMCs three times by centrifuging at 250× *g* for 10 min each time with 10 mL of sterile PBS.
6. Added 600 µL of RTL buffer with β-mercaptoethanol to the PBMCs, mixed, divided into two Eppendorf tubes, and frozen at −80 °C until RNA isolation.

### 2.4. Bio-Plex TGFβ Assays

Serum concentrations of TGF-β1, TGF-β2, and TGF-β3 were evaluated using a multiplexed bead-based immunoassay (Bio-Plex Pro™ TGF-β Assays, 3-Plex, catalog #: 10024984, Bio-Rad Laboratories, Inc., Hercules, CA, USA). The experiment was conducted according to the manufacturer’s instructions.

### 2.5. RNA Isolation

Total RNA was isolated from peripheral blood mononuclear cell (PBMC) samples, including T cells, B cells, NK cells, monocytes, and dendritic cells using the RNeasy Mini Kit (catalog #: 74104 Qiagen GmbH, Hilden, Germany) according to the protocol. The quantity and quality of the extracted RNA were assessed by measuring its absorbance using the NanoDrop One (Thermo Fisher Scientific, Madison, WI, USA). The RNA concentrations ranged between 58.9 and 250.3 ng/μL for the isolated PBMCs samples, with an A260/280 nm ratio between 1.7 and 2.0, which is considered acceptable. The obtained RNA samples were stored at −80 °C until further analysis.

### 2.6. Reverse Transcription

To obtain cDNA from RNA, the reverse transcription reaction was performed according to the manufacturer’s protocol using a High-Capacity cDNA Reverse Transcription kit with RNase Inhibitor (catalog #: 4374966 Applied Biosystems by Thermo Fisher Scientific, Vilnius, Lithuania). The final concentration of RNA in the reaction mixture in samples was equated to 0.5 μg/μL. The thermocycling reverse transcription parameters were as follows: 25 °C for 10 min, then 37 °C for 120 min, and 85 °C for 5 min. The cDNA samples were stored at −20 °C until further analysis.

### 2.7. Real-Time RT-PCR

A housekeeping *GAPDH* gene, encoding glyceraldehyde- 3-phosphate dehydrogenase (Hs_GAPHD_2-SG catalog #:QT01192646, QuantiTect^®^ Primer Assay, Qiagen, GMBh, Hilden, Germany) was used as the reference gene. The expression of the target genes, *TGFB1*, *TGFB2*, and *TGFB3* (Hs_TGFB1_1-SG catalog #:QT00000728; Hs_TGFB2_1-SG catalog #:QT00025718; Hs_TGFB3_1-SG catalog #:QT00001302, QuantiTect^®^ Primer Assay, Qiagen, GMBh, Hilden, Germany), was quantified using the QuantStudio^TM^ 5 Real-Time PCR Systems (Applied Biosystems by Thermo Fisher Scientific, Singapore) and PowerUp™ SYBR™ Green Master Mix (catalog #: A25741, Applied Biosystem by Thermo Fisher Scientific, **Waltham, MA, USA**). The reaction mixture contained 5 μL PowerUp™ SYBR™ Green Master Mix, 0.5 μL of each primer specific for the investigated gene, 4 μL of distilled water, and 1 μL of cDNA template. The final volume of each tube was 10 μL. The analysis was conducted in triplicates for each sample and carried out in separate tubes for reference and investigated genes. No template control (NTC) samples were included to ensure the quality of the assay in each trial. The real-time PCR conditions were as follows: UNG pretreatment for 2 min at 50 °C, PCR initial heat activation for 2 min at 95 °C, followed by 40 cycles of denaturation for 15 s at 95 °C, primer annealing for 30 s at 60 °C, and followed by elongation for 30 s at 72 °C. A post-amplification melting-curve analysis was performed in order to check the reaction specificity. The obtained Ct values were averaged. To estimate relative changes in the expression level, Δ values were calculated for the tested genes using the differences between the arithmetic mean of the Ct value obtained for the tested genes and the Ct for the reference gene (*GAPHD*) [40,41].

### 2.8. Statistical Analysis

The results were analyzed with the use of the STATISTICA 13.3 PL software (StatSoft Inc., Tulsa, OK, USA) and GraphPad Prism Version 8.4.3 (686) (GraphPad Software, San Diego, CA, USA). The Mann–Whitney U test was utilized to compare two independent samples, while Spearman’s rank correlation coefficient was used to assess the correlation between variables. Values for continuous variables are presented as median with 25th and 75th percentile. To assess the discriminative ability of TGF-β1, TGF-β3 concentration and Δ *TGFB1*, *TGFB3* expression in differentiating patients groups, a receiver operator characteristic (ROC) curve was constructed. The Youden index, which combines sensitivity and specificity, revealed an optimal cut-off point between these two factors for the parameters under investigation. Statistical significance was determined at a significance level of <0.05 for two-tailed *p*-values.

## 3. Results

### 3.1. Demographic Data and Results of Routine Laboratory Tests for Each Group

Among 32 patients, 24 patients had unruptured intracranial aneurysms (UIAs), including 18 women and 6 men, while 8 patients had ruptured intracranial aneurysms (RIAs), comprising 4 women and 4 men. The mean age of the IA group was 53 years (range: 24–72 years). Among the study participants, 13 individuals (41%) did not have a diagnosis of hypertension, 19 (59%) were hypertensive. Four patients (12%) in the study group were smokers; all of them had unruptured aneurysms. The control group consisted of 20 healthy volunteers, 14 females and 6 males, with a mean age of 51 years (range: 24–71 years). The analysis included patients with intracranial aneurysms (IAs), both unruptured (UIAs) and ruptured (RIAs), as well as individuals in the control (C) group. Table 1 presents aneurysm geometry characteristics (location, size, number, morphology), demographic data (sex, age), and laboratory test results for each group. In the IA group, significant differences were observed in several parameters compared to the control group, including white blood cells count (WBC), red blood cells count (RBC), hematocrit levels (HCT), prothrombin time (PT), international normalized ratio (INR), activated partial thromboplastin time (APTT), fibrinogen, and glucose levels. In the UIA group, significant differences were noted relative to the control group in RBC count, mean corpuscular volume (MCV), PT, INR, APTT, and, fibrinogen levels. The RIA group exhibited significant differences compared to the control group in WBC count, PT, INR, fibrinogen, K^+^, glucose, and urea levels. Furthermore, a direct comparison between RIA and UIA patients revealed significant differences in WBC count, MCV, APTT, and K^+^ levels.

### 3.2. Gene Expression Profiles of TGFB Factors in PBMCs: TGFB1 and TGFB3 as Indicators of Aneurysm Formation and Rupture

**Figure 1:**
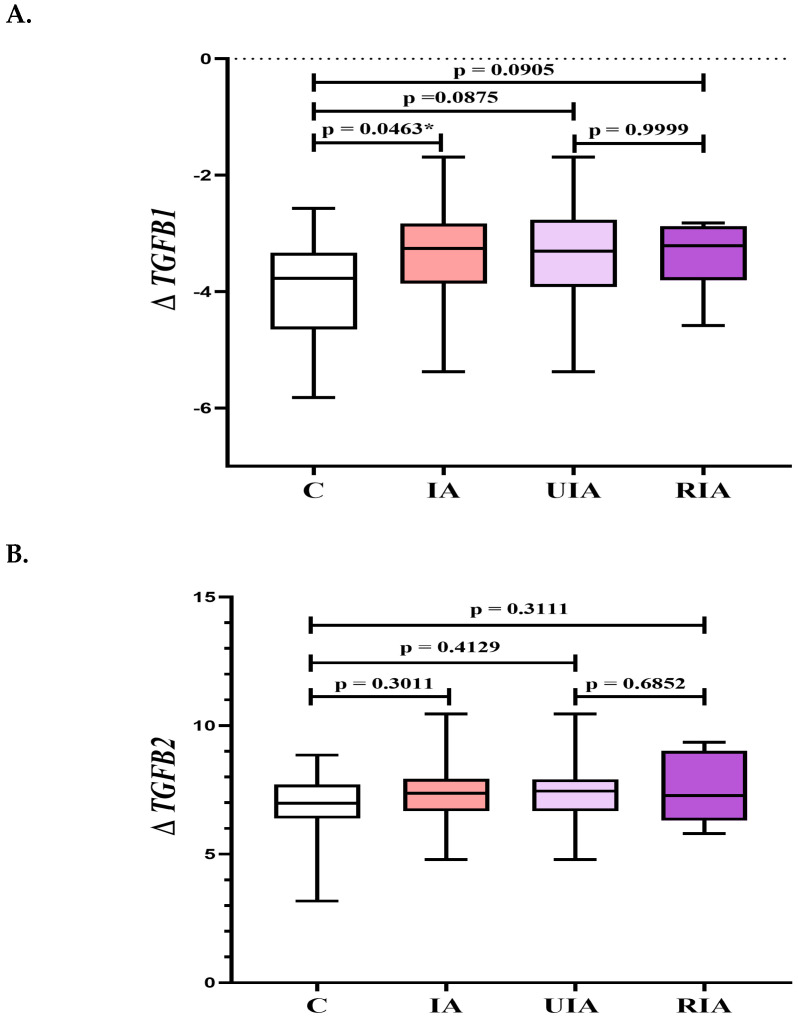

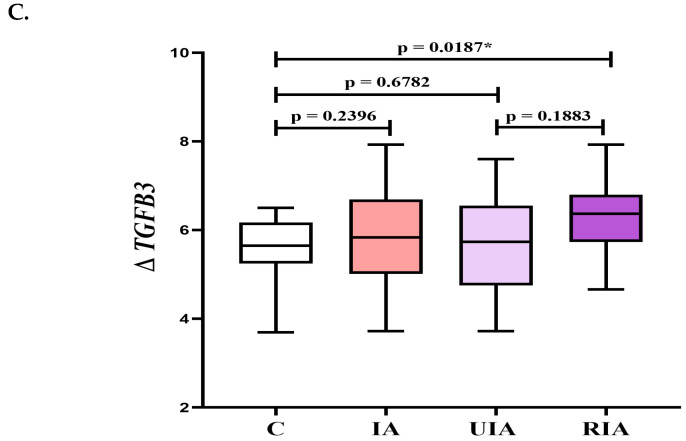
(**A–C**) The *TGFB1*, *TGFB2*, and *TGFB3* expression in PBMC samples in the intracranial aneurysm (IA) group, unruptured intracranial aneurysm (UIA) group, ruptured intracranial aneurysm (RIA) group, and the control group (C). Legend for Figure 1A–C *TGFB*, transforming growth factor β; Δ—differences between the arithmetic mean of the Ct value obtained for the tested genes and the Ct of the reference gene (*GAPHD*, gene—encoding glyceraldehyde-3-phosphate dehydrogenase); * *p*-Value of <0.05 was considered statistically significant.

The analysis of these growth factors was performed for the entire study group (IA = UIA + RIA) in comparison to healthy controls (C), and between subgroups (UIA vs. C and RIA vs. C, and UIA vs. RIA). The expression of *TGFB1* was significantly higher in the IA group compared to healthy controls in PBMC samples. No significant differences in *TGFB1* expression were observed in the remaining analyses (Figure 1A). Similarly, no significant differences in *TGFB2* expression were found between any of the analyzed groups (Figure 1B). The expression of *TGFB3* was significantly elevated in the RIA group compared to healthy controls in PBMC samples. The remaining analyses of *TGFB3* expression did not reveal statistically significant differences (Figure 1C).

### 3.3. Increased Serum TGF-β1 and TGF-β3 Concentrations as Promising Noninvasive Parameters for Assessing the Risk of Aneurysm Rupture

The serum concentration of TGF-β1 and TGF-β3 were significantly higher in the RIA group compared to the UIA group. The serum concentration of TGF-β2 was also higher in the RIA group compared to the UIA group, although not significantly. Detailed results of TGF-β1, TGF-β2, and TGF-β3 serum levels across individual subgroups are presented in Table 2.

### 3.4. Serum TGF-β1 and TGF-β3 Levels Are Associated with the Risk Factors for Intracranial Aneurysms

Serum concentrations of TGF-β1 were significantly higher in males than in females in the UIA group. Age also had an impact on TGF-β1 levels in UIA individuals. The serum concentration of TGF-β1 was significantly higher in patients aged <60 years than in those aged ≥60 years. TGF-β2 and TGF-β3 levels did not show significant differences based on sex or age (Table 3).

An increasing trend in the concentrations of TGF-β1 and TGF-β3 was observed with the size of the aneurysm. In contrast, TGF-β2 concentrations exhibited a decreasing trend. However, significant differences were noted only for TGF-β3, where patients with huge aneurysms (>10 mm) had significantly higher TGF-β3 concentrations in comparison to those with small aneurysms (<5 mm) (Table 3).

In the UIA group, serum TGF-β1 concentrations were significantly higher in patients with single aneurysms compared to those with multiple aneurysms. TGF-β2 and TGF-β3 levels did not show significant differences based on count of aneurysms (Table 3).

We did not observe significant differences in the serum concentrations of TGF-β factors based on aneurysm shape (saccular vs. polycyclic) in the UIA group. Additionally smoking and hypertension did not affect serum concentrations of TGF-β1, TGF-β2, and TGF-β3 (Table 3).

### 3.5. Correlation Coefficient Results in Unruptured Intracranial Aneurysm (UIA) Group

**Figure 2:**
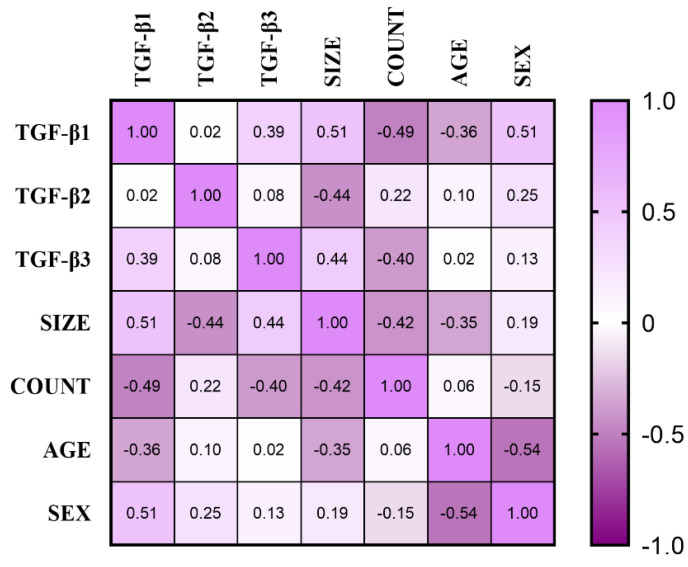
Heat maps of the complex relationship between serum concentrations of TGF-β1, TGF-β2, and TGF-β3, and variables such as aneurysm size, count, age, and sex in the UIA group. Legend of Figure 2. TGF-β, transforming growth factor β.

Heat maps illustrate the complex relationships between serum TGF-β1, TGF-β2, and TGF-β3 concentrations and variables such as aneurysm size, count, age, and sex in the UIA group (Figure 2). In the UIA group, we observed a significant positive correlation between serum TGF-β1 and aneurysm size (R = 0.515, *p* = 0.010), a negative correlation with aneurysm count (R = −0.492, *p* = 0.015), and a positive correlation with sex (R = 0.514, *p* = 0.010). Additionally, serum TGF-β3 exhibited a significant positive correlation with aneurysm size (R = 0.437, *p* = 0.033).

### 3.6. ROC Curve for TGFB1 and TGFB3 Expression and Concentrations

**Figure 3:**
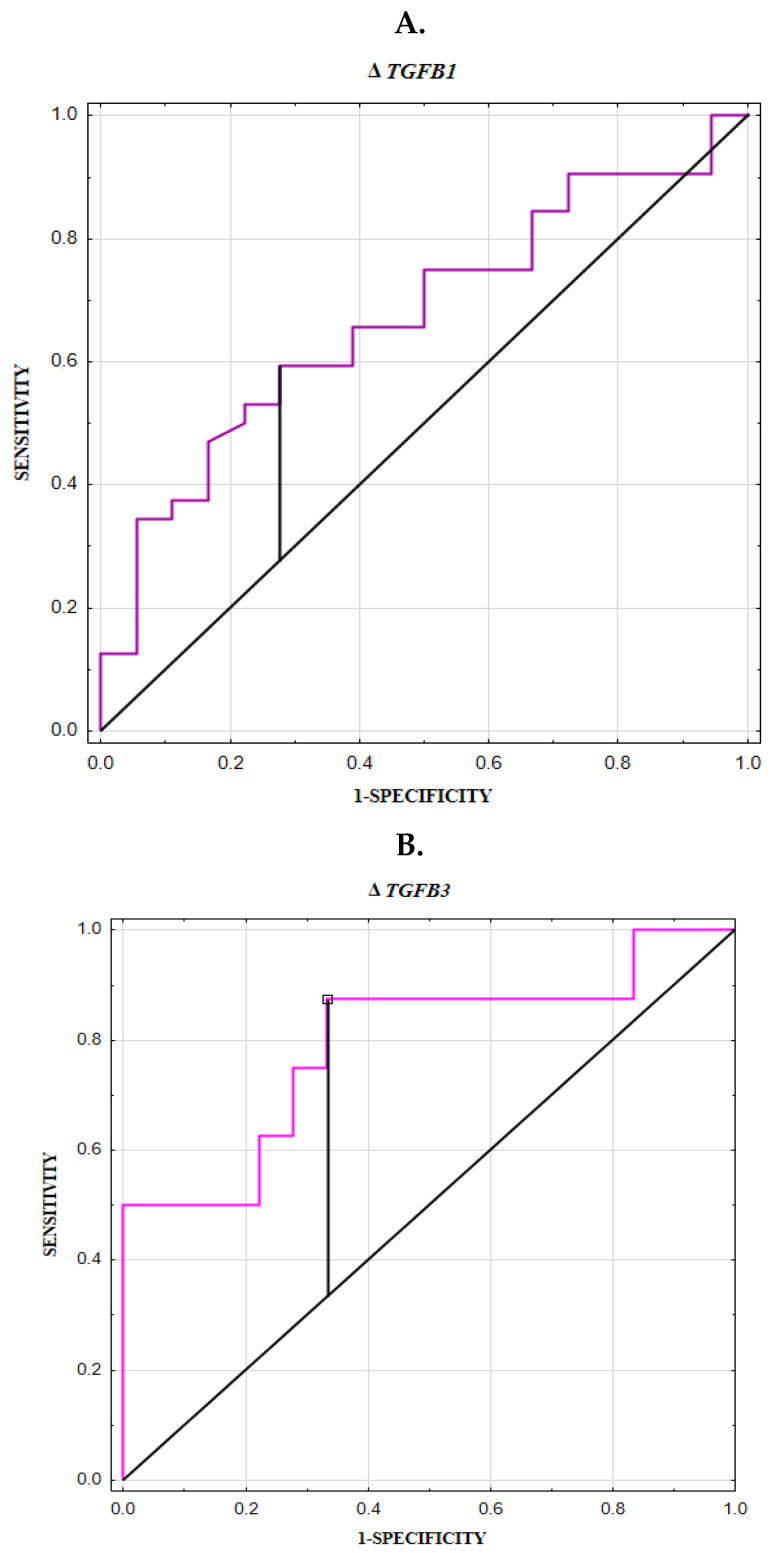

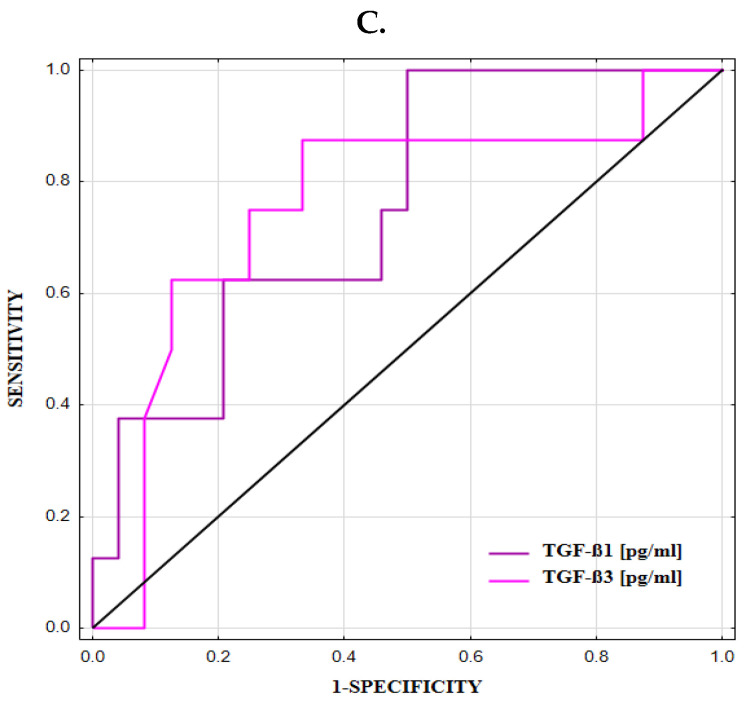
(**A**) ROC curve for *TGFB1* expression in identification of intracranial aneurysms. (**B**) ROC curve for *TGFB3* expression in identification of ruptured intracranial aneurysms. (**C**) ROC curve for serum TGF-β1 and TGF-β3 concentrations in differentiating ruptured aneurysms from unruptured.

*TGFB1* expression was diagnostically useful in identifying individuals with aneurysms, with an AUC of 0.671 (Table 4, Figure 3A). Moreover, *TGFB3* expression demonstrated diagnostic relevance in identifying ruptured aneurysms, with an AUC of 0.792 (Table 5, Figure 3B). TGF-β1 and TGF-β3 serum concentrations were useful for differentiating UIA patients from RIA individuals, with AUCs of 0.755 and 0.758, respectively (Table 6, Figure 3C).

## 4. Discussion

The current study demonstrated that *TGFB1* expression in PBMCs was significantly higher in the intracranial aneurysm group (comprising both UIA and RIA cases) compared to the control group. Further analysis revealed that patients with ruptured intracranial aneurysms (RIAs) exhibited significantly higher *TGFB3* expression in PBMCs compared to controls. Protein concentration analysis of the serum showed that levels of TGF-β1 and TGF-β3 were significantly elevated in the RIA group compared to the UIA group. Additionally, serum TGF-β1 levels were higher in men and in individuals under 60 years of age. Positive correlations were observed between serum levels of TGF-β1 and TGF-β3 and aneurysm size, with particularly high TGF-β3 levels in patients with giant aneurysms.

*TGFB1* gene expression in the PBMCs of intracranial aneurysm (IA) patients was significantly higher than in healthy individuals, suggesting that *TGFB1* may contribute to the molecular mechanisms underlying aneurysm formation. Moreover, *TGFB3* expression was significantly higher in PBMCs of patients with RIA compared to healthy controls, suggesting a potential role for *TGFB3* in aneurysm rupture. Additionally, we plotted the ROC curve for *TGFB1* expression, demonstrating its utility in identifying aneurysms (AUC = 0.671), whereas *TGFB3* expression was significantly effective in identifying ruptured aneurysms (AUC = 0.792). It should be emphasized that our study examined gene expression in PBMCs, not in vascular tissues, which has not been previously investigated. Evaluating gene expression in PBMCs provides novel insights into the involvement of inflammatory cells and *TGFB1* and *TGFB3* in aneurysm formation and rupture.

Supriya et al. demonstrated that mRNA expression levels of *TGFB1*, *TGFB2*, and *TGFB3* were elevated in aneurysm tissues from patients with ruptured aneurysms compared to control tissues; however, the difference for *TGFB3* was not statistically significant [23]. Based on the obtained results, the authors conclude that modulation of the TGF-β and MAPK signaling pathways in aneurysm patients may contribute to inflammatory responses, extracellular matrix (ECM) degradation, and apoptosis of vascular smooth muscle cells (SMCs) ultimately leading to vessel wall damage and potential rupture [23]. Several studies have linked the overactivation of the TGF-β pathway and mutations in the *TGFB3* ligand to significant involvement in cardiovascular conditions, including thoracic and abdominal aortic aneurysms, as observed in syndromes such as Marfan and Loeys–Dietz, as well as in arterial dissection and mitral valve disease [42,43,44,45,46]. In an experimental study, Dai et al. showed that active *TGFB1* overexpression through endovascular monogenic delivery or treatment with recombinant TGF-β1 protein leads to a reduction in proteolytic activity, mitigates wall degradation, facilitates tissue regeneration on the luminal surface, and contributes to the stabilization of abdominal aortic aneurysms [47]. Another perspective on the TGF-β pathway is presented by Soto et al., who observed reduced expression of *TGFB2* and its receptors, *TGFBR1* and *TGFBR2*, in patients with Marfan syndrome [48]. The authors emphasize that decreased gene expression may contribute to progressive aortic dilation, an increased risk of aneurysm formation, and aortic dissection. They propose that the chronic inflammatory state observed in these patients may paradoxically suppress key components of the TGF-β signaling pathway, thereby disrupting normal regulatory feedback mechanisms [48]. In contrast, Frösen et al. reported increased expression of *TGFBR2* and *TGFBR3* receptors in response to risk factors associated with saccular cerebral aneurysm rupture. Furthermore, they found that the absence of *TGFBR1* receptor expression impairs TGF-β1-mediated extracellular matrix synthesis, potentially promoting aneurysm growth [20]. It is also worth emphasizing that the loss of TGF-β pathway genes in endothelial cells of mice through inactivation of Smad2/3 [49] or Smad4 [32] causes vascular defects, highlighting the crucial role of TGF-β in endothelial cells. Other studies suggest that elevated expression of Smad3 following arterial injury transforms TGF-β1 into a stimulant of SMC proliferation. Thus, TGF-β, in the context of elevated Smad3, enhances canonical Wnt/β-catenin signaling, which promotes SMC proliferation [50].

In our study, *TGFB2* expression did not show significant differences among the analyzed groups; however, the lack of literature data on this isoform of TGF-β makes discussion concerning this topic impossible. The limited data available on *TGFB2* expression may result from its relatively low expression levels in vascular tissues compared to other TGF-β isoforms. Another possible reason for the scarcity of data is the research emphasis on the more prominent isoforms, *TGFB1* and *TGFB3*, which are more consistently implicated in inflammation and aneurysm rupture. Additionally, the functional redundancy among TGF-β isoforms may lead researchers to focus on the better-characterized signaling pathways. Studies using a rat carotid artery model have shown that *TGFB1* is the most dominantly expressed member of the TGF-β family at the mRNA level [51], and, in fact, it is the most frequently studied TGF-β isoform.

An additional key point is the dual role of TGF-β factors in vascular biology. On one hand, they support vascular structure and integrity by promoting collagen production. On the other hand, they can contribute to pathological vascular remodeling, which may ultimately lead to aneurysm rupture. This paradoxical function positions TGF-β as a pivotal factor in aneurysm development—capable of maintaining lesion stability over time while also serving as a key mediator of rupture. Summarizing, further research, particularly studies involving human samples from patients with unruptured and ruptured cerebral aneurysms, is essential to better understand the distinct roles of TGF-β isoforms in aneurysm formation and rupture. This is especially important given that current literature suggests these processes are driven by significantly different underlying mechanisms [25].

In our study, we also assessed the serum concentrations of TGF-β1, TGF-β2, and TGF-β3 in patients with unruptured and ruptured cerebral aneurysms, as well as in healthy controls. We found significantly higher levels of TGF-β1 and TGF-β3 in RIA patients compared to UIA patients. Additionally, the plotted ROC curves demonstrated the strong diagnostic value of these parameters in distinguishing ruptured aneurysms from unruptured ones (AUC for TGF-β1: 0.755, and for TGF-β3: 0.758). It can therefore be concluded that circulating concentrations of TGF-β could serve as promising noninvasive parameters for evaluating aneurysm stability or the risk of rupture. Nevertheless, further studies with larger cohorts are needed to establish appropriate cut-off values and to validate the diagnostic method, so that the assessment of blood TGF-β factor concentrations could be useful in making clinical decisions.

Supriya et al. showed that serum TGF-β levels were significantly elevated in patients with ruptured aneurysms compared to healthy individuals [23]. However, the authors did not specify which TGF-β factor was assessed, making it difficult to directly compare their findings with ours. Xu et al. suggest that TGF-β1 is involved in the formation of intracranial aneurysms, as its elevated levels—mediated by hypoxia-inducible factor 1-antisense RNA 1 (HIF1A-AS1)—may inhibit the proliferation of vascular smooth muscle cells [38]. Sathyan et al. reported substantially higher concentrations of TGF-β1 in the cerebrospinal fluid of patients with subarachnoid hemorrhage [52]. Hemorrhage is known to trigger an increase in leukocytes and platelets, which migrate to the rupture site and may act as significant sources of TGF-β factors [35].

In our analysis of routine test results, we found that the white blood cell count was significantly higher in the group with ruptured intracranial aneurysms compared to those with unruptured aneurysms. The infiltration of macrophages and their entrapment within the ECM exacerbate aortic damage through the increased release of TGF-β by the trapped macrophages [39,48]. Based on these findings, we hypothesize that leukocytes may contribute to the elevated blood TGF-β levels observed in our study, particularly due to their involvement in aneurysm formation and rupture.

Current analysis showed that the concentration of TGF-β1 positively correlated with sex. A detailed analysis revealed significantly higher serum TGF-β1 levels in men compared to women, despite the fact that the female sex is considered a predisposing factor for the formation and development of cerebral aneurysms and a higher risk of subarachnoid hemorrhage [53]. However, our results do not support an association between elevated TGF-β1 levels and female sex as a risk factor for aneurysm rupture; instead, they may align with previous studies suggesting a sex-regulated TGF-β1 system. In men, increased TGF-β1 levels may be related to lower circulating estrogen levels, whereas in women, the opposite is true [54]. Furthermore, co-modifying factors such as hypertension and sex have been associated with the *TGFB1* rs1800469 polymorphism, which may exacerbate brain aneurysms by promoting vascular ECM degradation [55]. Therefore, additional molecular studies are needed in this area.

Age is another crucial factor influencing the risk of aneurysm rupture. Our study demonstrated that individuals in the UIA group under 60 years old had significantly higher serum concentrations of TGF-β1 compared to those aged 60 years or older. This may reflect more effective TGF-β-dependent vessel repair mechanisms in younger individuals, contributing to long-term aneurysm stability [25]. In particular, histological analyses of aneurysm walls have revealed that thicker, intima-like structures are typically associated with unruptured aneurysms, particularly in younger individuals who possess more efficient repair mechanisms. In contrast, thinner, degenerated walls with hyaline deposits are more prone to rupture [24]. Therefore, further analyses of TGF-β concentrations should account for patient age and sex as potential confounding factors.

Additionally, we analyzed the association between aneurysm geometry factors—such as count and size—and TGF-β concentrations. We identified a significant negative correlation between serum TGF-β1 levels and aneurysm count. The further detailed analysis revealed that patients with single aneurysms exhibited significantly higher serum TGF-β1 concentrations compared to those with multiple aneurysms. This association may be influenced by the larger number of patients with single aneurysms in our cohort and requires further validation in a larger study population. Our study showed a significant positive correlation between serum TGF-β1, TGF-β3 levels and aneurysm size. For TGF-β3, we found significantly higher levels in patients with giant aneurysms compared to those with small aneurysms. Several studies have demonstrated that human abdominal aortic aneurysm dilation is accompanied by smooth muscle cell (SMC) apoptosis [56,57], and the resulting degenerated SMCs respond differently to TGF-β1 than healthy SMCs, contributing to changes in the cellular composition of the vascular wall [58]. These are noteworthy findings that show the importance of assessing TGF-β factors in the context of aneurysms size and the risk of rupture.

In summary, our findings reveal a correlation between elevated serum levels of TGF-β1 and TGF-β3 and key risk factors for cerebral aneurysm—such as sex, age, and aneurysm geometry. To date, no studies have systematically evaluated blood TGF-βs concentrations in relation to these risk factors, highlighting the novelty of our investigation and the need for further research in larger cohorts to establish their clinical relevance.

### Study Limitation

One limitation of our research is the relatively small sample size of the study groups, emphasizing the need for further investigation in a larger cohort to validate the promising and intriguing findings obtained. Another limitation is that the study included only patients with hemorrhage caused by ruptured aneurysms. Future research should also encompass patients with intracranial hemorrhage unrelated to the presence of cerebral aneurysms to provide a more comprehensive understanding The third limitation is that our study was conducted at a single center in a Polish population and included only middle-aged patients, which means that our results cannot be extrapolated to other ethnic groups or to younger patients, in whom RIA is more common; this warrants further research.

## 5. Conclusions

In summary, our study highlights that *TGFB1* expressed by PBMCs primarily contributes to aneurysm formation, while *TGFB3* is associated with aneurysm rupture. Evaluating gene expression in PBMCs offers novel insights into the involvement of inflammatory cells and *TGFBs* isoforms in aneurysm formation and rupture. Additionally, circulating levels of TGF-β1 and TGF-β3 may serve as valuable parameters for assessing aneurysm stability and the risk of rupture. Nevertheless, further research involving larger patient cohorts is necessary to establish definitive cut-off values and validate the clinical utility of blood TGF-β levels as a supportive tool for decision-making in the management of intracranial aneurysms. Analyses should account for patient age and sex as potential confounding factors.

## Figures and Tables

**Table 1:** Aneurysm geometry characteristics, demographic data, and basic laboratory parameters in the intracranial aneurysm (IA) group, unruptured intracranial aneurysm (UIA) group, and ruptured intracranial aneurysm (RIA) group, compared to the control group (C). A *p*-Value of <0.05 was considered statistically significant.

**Aneurysm Geometry Characteristics**								
	**IA Group** **No. (%)**		**UIA Group** **No. (%)**			**RIA Group** **No. (%)**		
**Aneurysm Location**								
MCA	3 (9%)		2 (8%)			1 (12.5%)		
ACA	1 (3%)		1 (4%)			-		
ICA	13 (41%)		12 (50%)			1 (12.5%)		
AComA	11 (34%)		5 (21%)			6 (75%)		
BA	4 (13%)		4 (17%)			-		
**Aneurysm size [mm]**								
<5 mm	9 (28%)		7 (29%)			2 (25%)		
5–10 mm	17 (53%)		12 (50%)			5 (62.5%)		
>10 mm	6 (19%)		5 (21%)			1 (12.5%)		
**Number of aneurysms**								
Single	22 (69%)		17 (71%)			5 (62.5%)		
Multiple	10 (31%)		7 (29%)			3 (37.5%)		
**Shape of aneurysms**								
Saccular	19 (59%)		14 (58%)			5 (62.5%)		
Polycyclic	13 (41%)		10 (42%)			3 (37.5%)		
**Demographic Data and Basic Laboratory Parameters**								
	**Median** (25th and 75th percentile)				**2-tailed *p*-Value**			
	**IA Group** (*n* = 32)	**UIA Group** (*n* = 24)	**RIA Group** (*n* = 8)	**Control Group** (*n* = 20)	**IA vs** **.** **C**	**UIA vs** **. C**	**RIA vs** **.** **C**	**UIA vs** **. RIA**
**Sex** female/male	**22/10**	**18/6**	**4/4**	**14/6**	0.9244	0.7102	0.5746	0.3784
**Age** **mean** (range)	**53** (24–72)	**54** (24–71)	**47** (26–72)	**51** (24–71)	0.5822	0.3086	0.5002	0.2927
**WBC** [×10^3^/µL]	**7.19** (5.86–8.877)	**6.48** (5.46–7.96)	**9.79** (7.23–15.33)	**5.73** (5.19–6.76)	**0.0118**	0.1018	**0.0007**	**0.0065**
								
**RBC** [×10^6^/µL]	**4.54** (4.20–4.77)	**4.53** (4.20–4.77)	**4.63** (4.04–4.79)	**4.71** (4.36–5.18)	**0.0369**	**0.0483**	0.1816	0.8814
**HGB** [g/dL]	**13.5** (12.8–14.4)	**13.5** (12.8–14.4)	**13.7** (12.2–14.7)	**14.0** (13.1–14.6)	0.3551	0.3555	0.6358	0.9490
**HCT** [%]	**38.9** (37.4–42.4)	**38.8** (37.6–42.6)	**39.5** (34.8–41.8)	**40.8** (38.9–43.9)	**0.0487**	0.0832	0.1226	0.5641
**MCV** [fl]	**87.7** (85.60–91.30)	**89.6** (86.5–91.9)	**86.0** (83.7–86.4)	**86.6** (85.8–88.3)	0.2347	**0.0339**	0.1991	**0.0328**
**PLT** [×10^3^/µL]	**227** (193–252)	**217** (191–248)	**242** (222–279)	**239** (218–290)	0.1214	0.0605	0.9009	0.2197
**PCT** [%]	**0.23** (0.21–0.26)	**0.23** (0.21–0.25)	**0.24** (0.22–0.27)	**0.25** (0.23–0.30)	0.1625	0.1355	0.5661	0.5935
**MPV** [fl]	**10.3** (9.9–12.0)	**10.4** (10.1–11.2)	**9.9** (9.4–10.5)	**10.2** (9.8–10.7)	0.3173	0.1177	0.5327	0.1035
**PDW** [fl]	**11.8** (10.90–13.40)	**12.1** (11.2–13.7)	**11.0** (9.6–12.4)	**11.4** (10.9–12.2)	0.2499	0.0832	0.5327	0.1603
**P-LCR** [%]	**26.9** (23.7–32.9)	**27.8** (25.0–34.6)	**23.4** (20.0–29.1)	**25.2** (22.7–31.2)	0.2347	0.0790	0.5661	0.1241
**PT** [s]	**14.3** (13.6–14.8)	**14.3** (13.8–14.7)	**13.9** (13.3–15.2)	**13.0** (12.8–13.9)	**0.0003**	**0.0003**	**0.0428**	0.6540
**INR**	**1.06** (1.03–1.11)	**1.07** (1.04–1.10)	**1.03** (1.02–1.14)	**0.98** (0.98–1.01)	**<0.0001**	**<0.0001**	**0.0047**	0.7816
**APTT** [s]	**30.0** (27.1–31.6)	**30.7** (28.0–32.1)	**27.5** (25.6–29.2)	**26.8** (25.8–28.6)	**0.0074**	**0.0006**	0.9801	**0.0462**
**Fibrinogen** [mg/dL]	**305** (268–361)	**302** (265–361)	**309** (284–360)	**262** (250–300)	**0.0118**	**0.0339**	**0.0285**	0.5355
**Na^+^** [mmol/L]	**140** (139–142)	**140** (139–142)	**141** (139–142)	**140** (139–141)	0.3173	0.4617	0.2806	0.7169
**K^+^** [mmol/L]	**4.1** (3.9–4.5)	**4.2** (4.0–4.6)	**3.6** (3.1–4.3)	**4.4** (4.2–4.6)	0.0510	0.2011	**0.0114**	**0.0368**
**Glucose** [mg/dL]	**102** (91–130)	**98** (91–125)	**122** (94–155)	**94** (89–100)	**0.0351**	0.1069	**0.0247**	0.3345
**Urea** [mg/dL]	**29.96** (21.40–36.38)	**27.82** (21.40–36.38)	**29.96** (29.64–34.24)	**25.03** (21.90–28.03)	0.0753	0.2183	**0.0328**	0.6540
**Creatinine** [mg/dL]	**0.71** (0.62–0.83)	**0.71** (0.64–0.82)	**0.70** (0.54–0.87)	**0.73** (0.68–0.81)	0.4841	0.5360	0.5661	0.9830
**eGFR** [mL/min]	**104** (84–123)	**101** (84–121)	**108** (89–137)	**101** (93–112)	0.8595	0.9164	0.4688	0.4284

Legend for Table 1: ACA, distal anterior cerebral artery; AComA, anterior communicating artery; APTT, activated partial thromboplastin; BA, basilar artery; eGFR, estimated glomerular filtration rate; HCT, hematocrit; HGB, hemoglobin; IA, intracranial aneurysm; ICA, interial cartoid artery; INR, international normalized ratio; K^+^, potassium ion; MCA, middle cerebral artery; MCV, mean corpuscular volume; MPV, mean platelet volume; Na^+^, sodium ion; PCT, plateletcrit; PDW, platelet distribution width; P-LCR, platelet—large cell ratio; PLT, platelet count, PT, prothrombin time; UIA, unruptured intracranial aneurysm; RIA, ruptured intracranial aneurysm; RBC, red blood cell count; WBC, white blood cell count. Conversion factors to SI units are as follows: for WBC—1.0, for RBC—1.0, for HGB—10.0, for PLT—1.0, for glucose—0.0555, for creatinine—88.

**Table 2:** The serum concentration of TGF-β1, TGF-β2, and TGF-β3 in the intracranial aneurysm (IA), unruptured intracranial aneurysm (UIA), ruptured intracranial aneurysm (RIA) group, and control group (C).

Parameter [pg/mL]	Median (25th and 75th Percentile)				2-Tailed *p*-Value			
	IA Group (*n* = 32)	UIA Group (*n* = 24)	RIA Group (*n* = 8)	C Group (*n* = 20)	IA vs. C	UIA vs. C	RIA vs. C	UIA vs. RIA
**TGF-β1**	**48,466** (38,597–60,862)	**45,206** (37,359–56,918)	**60,183** (46,060–65,440)	**52,784** (38,485–61,275)	0.7024	0.3199	0.2806	0.0328 *
**TGF-β2**	**1108** (964–1318)	**1048** (960–1288)	**1274** (1111–1343)	**1017** (942–1222)	0.3575	0.7751	0.0625	0.1241
**TGF-β3**	**441** (403–490)	**418** (393–466)	**491** (449–502)	**453** (399–517)	0.3196	0.1220	0.5410	0.0301 *

Legend for Table 2: TGF-β, transforming growth factor beta; IA, intracranial aneurysm; UIA, unruptured intracranial aneurysm; RIA, ruptured intracranial aneurysm; C, control, *—the 2-tailed *p*-value of <0.05 is considered statistically significant.

**Table 3:** Serum concentrations of TGF-β1, TGF-β2, and TGF-β3 depending on the risk factors (sex, age, size, count, shape of aneurysms, smoking, and hypertension) in unruptured intracranial aneurysms patients (UIA).

**Parameter** **[pg/mL]**	**Sex**			**2-Tailed *p*-Value**
	**Female** *n* = 18	**Male** *n* = 6		
**TGF-β1**	**43,448** (35,530–50,411)	**57,870** (55,473–63,605)		**0.0118** *
**TGF-β2**	**991** (942–1250)	**1174** (1047–1300)		0.2509
**TGF-β3**	**418** (389–454)	**416** (408–470)		0.5805
	**Age**			**2-Tailed *p*-Value**
	**< 60 years** *n* = 12	**≥ 60 years** *n* = 12		
**TGF-β1**	**56,846** (43,177–62,751)	**40,887** (37,461–45,206)		0.0205 *
**TGF-β2**	**1005** (937–1276)	**1052** (983–11,288)		0.4776
**TGF-β3**	**418** (386–462)	**424** (396–461)		0.9778
	**Size of Aneurysms**			**2-Tailed *p*-Value**
	**<5 mm** *n* = 7	**5–10 mm** *n* = 12	**>10 mm** *n* = 5	**<5 mm vs. 5–10 mm** **<5 mm vs. >10 mm** **5–10 mm vs. >10 mm**
**TGF-β1**	**37,257** (34,543–48,961)	**45,206** (38,778–53,149)	**61,027** (56,991–64,225)	0.2268 0.1490 0.0818
**TGF-β2**	**1250** (993–1359)	**1019** (967–1133)	**942** (932–1252)	0.1956 0.1490 0.5743
**TGF-β3**	**408** (364–419)	**426** (396–449)	**470** (420–508)	0.4320 0.0303 * 0.1037
	**Count of Aneurysms**			**2-Tailed *p*-Value**
	**Single** *n* = 17	**Multiple** *n* = 7		
**TGF-β1**	**50,411** (42,635–56,991)	**35,530** (29,885–42,969)		**0.0337** *
**TGF-β2**	**1049** (961–1250)	**1047** (960–1351)		0.5761
**TGF-β3**	**438** (408–477)	**408** (364–417)		0.0645
	**Shape of Aneurysms**			
	**Saccular** *n* = 14	**Polycyclic** *n* = 10		**2-Tailed *p*-Value**
**TGF-β1**	**45,206** (37,257–56,991)	**46,954** (38,416–50,825)		0.9771
**TGF-β2**	**1097** (961–1252)	**1012** (960–1300)		0.7521
**TGF-β3**	**422** (391–454)	**430** (408–470)		0.7521
	**Smoking**			**2-Tailed *p*-Value**
	**No** *n* = 20	**Yes** *n* = 4		
**TGF-β1**	**45,206** (38,041–56,846)	**43,834** (33,571–58,930)		0.8519
**TGF-β2**	**1018** (951–1199)	**1289** (1123–1346)		0.0969
**TGF-β3**	**418** (405–449)	**419** (338–504)		0.9701
	**Hypertension**			**2-Tailed *p*-Value**
	**No** *n* = 10	**Yes** *n* = 14		
**TGF-β1**	**53,149** (37,666–61,027)	**42,802** (37,257–50,411)		0.3407
**TGF-β2**	**1083** (961–1253)	**1020** (942–1326)		0.2591
**TGF-β3**	**411** (364–470)	**419** (408–445)		0.4031

Legend for Table 3: TGF-β, transforming growth factor beta, Results are presented as median (25th and 75th percentile), *—the 2-Tailed *p*-Value of <0.05 is considered statistically significant.

**Table 4:** Diagnostic utility of *TGFB1* expression in patients with intracranial aneurysms (IAs) compared to healthy controls (C).

	Cut-Off	Youden Index	AUC ± SE	Se [%]	Sp [%]	PPV [%]	NPV [%]	ACC [%]	*p*-Value
**Δ** ***TGFB1***	−3.412	0.32	0.671 ±0.077	60	72	79	50	64	**0.0267** *

Legend for Table 4: ACC, diagnostic accuracy; AUC, area under the ROC curve; Cut-off (based on the highest Youden index); NPV, negative predictive value; PPV, positive predictive value; Se, diagnostic sensitivity; SE, standard error; Sp, diagnostic specificity; *—the 2-Tailed *p*-Value of <0.05 is considered statistically significant.

**Table 5:** Diagnostic utility of *TGFB3* expression in patients with ruptured intracranial aneurysms (RIAs) compared to healthy controls (C).

	Cut-Off	Youden Index	AUC ± SE	Se [%]	Sp [%]	PPV [%]	NPV [%]	ACC [%]	*p*-Value
**Δ** ***TGFB3***	5.683	0.54	0.792 ± 0.106	88	67	54	54	73	**0.0061** *

Legend for Table 5: ACC, diagnostic accuracy; AUC, area under the ROC curve; Cut-off (based on the highest Youden index); NPV, negative predictive value; PPV, positive predictive value; Se, diagnostic sensitivity; SE, standard error; Sp, diagnostic specificity; *—the 2-Tailed *p*-Value of <0.05 is considered statistically significant.

**Table 6:** Diagnostic utility of TGF-β1, TGF-β3 in differentiating unruptured with ruptured intracranial aneurysms.

	Cut-Off	Youden Index	AUC ± SE	Se [%]	Sp [%]	PPV [%]	NPV [%]	ACC [%]	*p*-Value
**TGF-β1 [pg/mL]**	44,973	0.50	0.755 ± 0.092	100	50	40	100	63	**0.0056** *
**TGF-β3 [pg/mL]**	444	0.54	0.758 ± 0.106	86	67	47	94	72	**0.0150** *

Legend for Table 6: ACC, diagnostic accuracy; AUC, area under the ROC curve; TGF-β, transforming growth factor beta; Cut-off (based on the highest Youden index); NPV, negative predictive value; PPV, positive predictive value; Se, diagnostic sensitivity; SE, standard error; Sp, diagnostic specificity; *—the 2-Tailed *p*-Value of <0.05 is considered statistically significant.

## Data Availability

The datasets generated and/or analyzed during the current study are not publicly available, but are available from the corresponding author (J.K.) on reasonable request.

## Abbreviations

ACC	diagnostic accuracy
APTT	activated partial thromboplastin time
AUC	area under the ROC curve
cDNA	complementary DNA
Cut-off	(cut-off based on the highest Youden index)
ECs	endothelial cells
EEL	external elastic lamina
GAPDH	glyceraldehyde 3-phosphate dehydrogenase
HIF1A-AS1	hypoxia-inducible factor 1α-antisense RNA
IA	intracranial aneurysm
IEL	internal elastic lamina
INR	international normalized ratio
K+	potassium ion
MCV	mean corpuscular volume
mRNA	messenger RNA
NADPH	nicotinamide adenine dinucleotide phosphate
NOX4	NADPH oxidase 4
NPV	negative predictive value
PBMCs	peripheral blood mononuclear cells
PPV	positive predictive value
PT	prothrombin time
RBC	red blood cell count
RIA	ruptured intracranial aneurysm
RNA	ribonucleid acid
SAH	subarachnoid hemorrhage
Se	diagnostic sensitivity
SE	standard error
Smad	similar to mother against decapentaplegic
SMCs	smooth muscle cells
Sp	diagnostic specificity
SPARC	secreted protein acidic and rich in cysteine
TGF-β	transforming growth factor β
TβR	TGF-β receptor
UIA	unruptured intracranial aneurysm
WBC	white blood cell count

## References

[1] Ingall T., Asplund K., Mähönen M., Bonita R. (2000). A multinational comparison of subarachnoid hemorrhage epidemiology in the WHO MONICA stroke study. Stroke.

[2] Østbye T., Levy A.R., Mayo N.E. (1997). Hospitalization and Case-Fatality Rates for Subarachnoid Hemorrhage in Canada from 1982 Through 1991. Stroke.

[3] Xu Z., Rui Y.-N., Hagan J.P., Kim D.H. (2019). Intracranial Aneurysms: Pathology, Genetics, and Molecular Mechanisms. NeuroMolecular Med..

[4] Etminan N., Rinkel G.J. (2016). Unruptured intracranial aneurysms: Development, rupture and preventive management. Nat. Rev. Neurol..

[5] Shih S.-C., Ju M., Liu N., Mo J.-R., Ney J.J., Smith L.E.H. (2003). Transforming growth factor β1 induction of vascular endothelial growth factor receptor 1: Mechanism of pericyte-induced vascular survival in vivo. Proc. Natl. Acad. Sci. USA.

[6] Song Y., Liu P., Li Z., Shi Y., Huang J., Li S., Liu Y., Zhang Z., Wang Y., Zhu W. (2018). The Effect of Myosin Light Chain Kinase on the Occurrence and Development of Intracranial Aneurysm. Front. Cell. Neurosci..

[7] Kamińska J., Lyson T., Chrzanowski R., Sawicki K., Milewska A.J., Tylicka M., Zińczuk J., Matowicka-Karna J., Dymicka-Piekarska V., Mariak Z. (2020). Ratio of IL-8 in CSF Versus Serum Is Elevated in Patients with Unruptured Brain Aneurysm. J. Clin. Med..

[8] Kamińska J., Dymicka-Piekarska V., Chrzanowski R., Sawicki K., Milewska A.J., Zińczuk J., Tylicka M., Jadeszko M., Mariak Z., Kratz E.M. (2021). IL-6 Quotient (The Ratio of Cerebrospinal Fluid IL-6 to Serum IL-6) as a Biomarker of an Unruptured Intracranial Aneurysm. J. Inflamm. Res..

[9] Kamińska J., Maciejczyk M., Ćwiklińska A., Matowicka-Karna J., Koper-Lenkiewicz O.M. (2022). Pro-Inflammatory and Anti-Inflammatory Cytokines Levels are Significantly Altered in Cerebrospinal Fluid of Unruptured Intracranial Aneurysm (UIA) Patients. J. Inflamm. Res..

[10] Rodrigues V.J., Elsayed S., Loeys B.L., Dietz H.C., Yousem D.M. (2009). Neuroradiologic Manifestations of Loeys-Dietz Syndrome Type 1. Am. J. Neuroradiol..

[11] Vanakker O.M., Hemelsoet D., De Paepe A. (2011). Hereditary Connective Tissue Diseases in Young Adult Stroke: A Comprehensive Synthesis. Stroke Res. Treat..

[12] Kim S.T., Brinjikji W., Kallmes D.F. (2016). Prevalence of Intracranial Aneurysms in Patients with Connective Tissue Diseases: A Retrospective Study. Am. J. Neuroradiol..

[13] Mattson M.P., Barger S.W., Furukawa K., Bruce A.J., Wyss-Coray T., Mark R.J., Mucke L. (1997). Cellular signaling roles of TGFβ, TNFα and βAPP in brain injury responses and Alzheimer’s disease. Brain Res. Rev..

[14] Agrawal S.K. (2013). Aneurysm embolization with biologically active coils: An animal study. Neurol. Res..

[15] Li M.O., Wan Y.Y., Sanjabi S., Robertson A.-K.L., Flavell R.A. (2006). Transforming growth factor-β regulation of immune responses. Annu. Rev. Immunol..

[16] de Caestecker M. (2004). The transforming growth factor-β superfamily of receptors. Cytokine Growth Factor Rev..

[17] Hata A., Chen Y.G. (2016). TGF-β signaling from receptors to smads. Cold Spring Harb. Perspect. Biol..

[18] Ruiz-Ortega M., Rodríguez-Vita J., Sanchez-Lopez E., Carvajal G., Egido J. (2007). TGF-β signaling in vascular fibrosis. Cardiovasc. Res..

[19] Rotzer D., Roth M., Lutz M., Lindemann D., Sebald W., Knaus P. (2001). Type III TGF-β receptor-independent signalling of TGFβ2 via TβRII-B, an alternatively spliced TGF-β type II receptor. EMBO J..

[20] Frösen J., Piippo A., Paetau A., Kangasniemi M., Niemelä M., Hernesniemi J., Jääskeläinen J. (2006). Growth factor receptor expression and remodeling of saccular cerebral artery aneurysm walls: Implications for biological therapy preventing rupture. Neurosurgery.

[21] Tsai S., Hollenbeck S.T., Ryer E.J., Edlin R., Yamanouchi D., Kundi R., Wang C., Liu B., Kent K.C. (2009). TGF-β through Smad3 signaling stimulates vascular smooth muscle cell proliferation and neointimal formation. Am. J. Physiol. Circ. Physiol..

[22] Vincze C., Pál G., Wappler E.A., Szabó É.R., Nagy Z.G., Lovas G., Dobolyi A. (2010). Distribution of mRNAs encoding transforming growth factors-β1, -2, and -3 in the intact rat brain and after experimentally induced focal ischemia. J. Comp. Neurol..

[23] Supriya M., Christopher R., Devi B.I., Bhat D.I., Shukla D., Kalpana S.R. (2022). Altered MicroRNA Expression in Intracranial Aneurysmal Tissues: Possible Role in TGF-β Signaling Pathway. Cell. Mol. Neurobiol..

[24] Kataoka K., Taneda M., Asai T., Kinoshita A., Ito M., Kuroda R. (1999). Structural Fragility and Inflammatory Response of Ruptured Cerebral Aneurysms. Stroke.

[25] Frösen J., Piippo A., Paetau A., Kangasniemi M., Niemelä M., Hernesniemi J., Jääskeläinen J. (2004). Remodeling of Saccular Cerebral Artery Aneurysm Wall Is Associated with Rupture. Stroke.

[26] Travis M.A., Sheppard D. (2014). TGF-β Activation and Function in Immunity. Annu. Rev. Immunol..

[27] Liu Z., Ajimu K., Yalikun N., Zheng Y., Xu F. (2019). Potential Therapeutic Strategies for Intracranial Aneurysms Targeting Aneurysm Pathogenesis. Front. Neurosci..

[28] Frangogiannis N.G. (2017). The role of transforming growth factor (TGF)-β in the infarcted myocardium. J. Thorac. Dis..

[29] Majesky M.W., Lindner V., Twardzik D.R., Schwartz S.M., Reidy M.A. (1991). Production of transforming growth factor β1 during repair of arterial injury. J. Clin. Investig..

[30] Chen C.-R., Kang Y., Siegel P.M., Massagué J. (2002). E2F4/5 and p107 as Smad Cofactors Linking the TGFβ Receptor to c-myc Repression. Cell.

[31] Siegel P.M., Massagué J. (2003). Cytostatic and apoptotic actions of TGF-β in homeostasis and cancer. Nat. Rev. Cancer.

[32] Crist A.M., Lee A.R., Patel N.R., Westhoff D.E., Meadows S.M. (2018). Vascular deficiency of Smad4 causes arteriovenous malformations: A mouse model of Hereditary Hemorrhagic Telangiectasia. Angiogenesis.

[33] Santiago-Sim T., Mathew-Joseph S., Pannu H., Milewicz D.M., Seidman C.E., Seidman J.G., Kim D.H. (2009). Sequencing of TGF-β Pathway Genes in Familial Cases of Intracranial Aneurysm. Stroke.

[34] Frösen J. (2014). Smooth Muscle Cells and the Formation, Degeneration, and Rupture of Saccular Intracranial Aneurysm Wall—A Review of Current Pathophysiological Knowledge. Transl. Stroke Res..

[35] Frösen J., Cebral J., Robertson A.M., Aoki T. (2019). Flow-induced, inflammation-mediated arterial wall remodeling in the formation and progression of intracranial aneurysms. Neurosurg. Focus.

[36] Nanjo H., Sho E., Komatsu M., Sho M., Zarins C.K., Masuda H. (2006). Intermittent short-duration exposure to low wall shear stress induces intimal thickening in arteries exposed to chronic high shear stress. Exp. Mol. Pathol..

[37] Darsaut T., Salazkin I., Ogoudikpe C., Gevry G., Bouzeghrane F., Raymond J. (2006). Effects of stenting the parent artery on aneurysm filling and gene expression of various potential factors involved in healing of experimental aneurysms. Interv. Neuroradiol..

[38] Xu J., Zhang Y., Chu L., Chen W., Du Y., Gu J. (2018). Long non-coding RNA HIF1A-AS1 is upregulated in intracranial aneurysms and participates in the regulation of proliferation of vascular smooth muscle cells by upregulating TGF-β1. Exp. Ther. Med..

[39] Plana E., Oto J., Medina P., Fernández-Pardo Á., Miralles M. (2020). Novel contributions of neutrophils in the pathogenesis of abdominal aortic aneurysm, the role of neutrophil extracellular traps: A systematic review. Thromb. Res..

[40] Livak K.J., Schmittgen T.D. (2001). Analysis of Relative Gene Expression Data Using Real-Time Quantitative PCR and the 2−ΔΔCT Method. Methods.

[41] Pietrzak J., Świechowski R., Wosiak A., Wcisło S., Balcerczak E. (2024). ADAMTS Gene-Derived circRNA Molecules in Non-Small-Cell Lung Cancer: Expression Profiling, Clinical Correlations and Survival Analysis. Int. J. Mol. Sci..

[42] Bertoli-Avella A.M., Gillis E., Morisaki H., Verhagen J.M.A., De Graaf B.M., Van De Beek G., Gallo E., Kruithof B.P.T., Venselaar H., Myers L.A. (2015). Mutations in a TGF-β ligand, TGFB3, cause syndromic aortic aneurysms and dissections. J. Am. Coll. Cardiol..

[43] Tingting T., Wenjing F., Qian Z., Hengquan W., Simin Z., Zhisheng J., Shunlin Q. (2020). The TGF-β pathway plays a key role in aortic aneurysms. Clin. Chim. Acta.

[44] Hara H., Takeda N., Fujiwara T., Yagi H., Maemura S., Kanaya T., Nawata K., Morita H., Komuro I. (2019). Activation of TGF-β signaling in an aortic aneurysm in a patient with Loeys-Dietz syndrome caused by a novel loss-of-function variant of TGFBR1. Hum. Genome Var..

[45] Takeda N., Hara H., Fujiwara T., Kanaya T., Maemura S., Komuro I. (2018). TGF-β signaling-related genes and thoracic aortic aneurysms and dissections. Int. J. Mol. Sci..

[46] Chen J., Chang R. (2022). Association of TGF-β Canonical Signaling-Related Core Genes with Aortic Aneurysms and Aortic Dissections. Front. Pharmacol..

[47] Dai J., Losy F., Guinault A.-M., Pages C., Anegon I., Desgranges P., Becquemin J.-P., Allaire E. (2005). Overexpression of Transforming Growth Factor-β1 Stabilizes Already-Formed Aortic Aneurysms. Circulation.

[48] Soto M.E., Rodríguez-Brito M., Pérez-Torres I., Herrera-Alarcon V., Martínez-Hernández H., Hernández I., Castrejón-Téllez V., Peña-Ocaña B.A., Alvarez-Leon E., Manzano-Pech L. (2025). Analysis of FBN1, TGFβ2, TGFβR1 and TGFβR2 mRNA as Key Molecular Mechanisms in the Damage of Aortic Aneurysm and Dissection in Marfan Syndrome. Int. J. Mol. Sci..

[49] Itoh F., Itoh S., Adachi T., Ichikawa K., Matsumura Y., Takagi T., Festing M., Watanabe T., Weinstein M., Karlsson S. (2012). Smad2/Smad3 in endothelium is indispensable for vascular stability via S1PR1 and N-cadherin expressions. Blood.

[50] DiRenzo D.M., Chaudhary M.A., Shi X., Franco S.R., Zent J., Wang K., Guo L.-W., Kent K.C. (2016). A crosstalk between TGF-β/Smad3 and Wnt/β-catenin pathways promotes vascular smooth muscle cell proliferation. Cell. Signal..

[51] Ryan S.T., Koteliansky V.E., Gotwals P.J., Lindner V. (2003). Transforming Growth Factor-Beta-Dependent Events in Vascular Remodeling following Arterial Injury. J. Vasc. Res..

[52] Flood C., Akinwunmi J., Lagord C., Daniel M., Berry M., Jackowski A., Logan A. (2001). Transforming Growth Factor-β1 in the Cerebrospinal Fluid of Patients with Subarachnoid Hemorrhage: Titers Derived from Exogenous and Endogenous Sources. J. Cereb. Blood Flow Metab..

[53] de Rooij N.K., Linn F.H.H., van der Plas J.A., Algra A., Rinkel G.J.E. (2007). Incidence of subarachnoid haemorrhage: A systematic review with emphasis on region, age, gender and time trends. J. Neurol. Neurosurg. Psychiatry.

[54] Recouvreux M.V., Lapyckyj L., Camilletti M.A., Guida M.C., Ornstein A., Rifkin D.B., Becu-Villalobos D., Díaz-Torga G. (2013). Sex Differences in the Pituitary Transforming Growth Factor-β1 System: Studies in a Model of Resistant Prolactinomas. Endocrinology.

[55] Sathyan S., Koshy L.V., Srinivas L., Easwer H.V., Premkumar S., Nair S., Bhattacharya R.N., Alapatt J.P., Banerjee M. (2015). Pathogenesis of intracranial aneurysm is mediated by proinflammatory cytokine TNFA and IFNG and through stochastic regulation of IL10 and TGFB1 by comorbid factors. J. Neuroinflammation.

[56] Sho E., Sho M., Nanjo H., Kawamura K., Masuda H., Dalman R.L. (2005). Comparison of cell-type-specific vs transmural aortic gene expression in experimental aneurysms. J. Vasc. Surg..

[57] Henderson E.L., Geng Y.-J., Sukhova G.K., Whittemore A.D., Knox J., Libby P. (1999). Death of Smooth Muscle Cells and Expression of Mediators of Apoptosis by T Lymphocytes in Human Abdominal Aortic Aneurysms. Circulation.

[58] Fukui D., Miyagawa S., Soeda J., Tanaka K., Urayama H., Kawasaki S. (2003). Overexpression of transforming growth factor β1 in smooth muscle cells of human abdominal aortic aneurysm. Eur. J. Vasc. Endovasc. Surg..

